# Carbonate of Ammonia in Squamous Affections of the Skin

**Published:** 1849-01

**Authors:** 


*Carbonate of Ammonia in Squamous Affections of the Skin.* By M. Cazenave.—In psoriasis, and other scaly diseases of the skin, M. Cazenave has obtained considerable benefit from the internal employment of this remedy. He gives it in the form of syrup, in doses of from 6 to 30 grains daily. The physiological effects are, complete anorexia, general debility, rapid emaciation, occasional diarrhœa, and some febrile excitement of the pulse; the countenance becomes paler. Some patients were obliged to discontinue the use of the drug, on account of the severity of these symptoms.—*Monthly Journal from, Gaz. des Hôp.*, Sept. 14.

